# Integrated Morphological, Molecular, and Chemical Characterization of the Macrofungus *Pholiota Gallica* With Phylogenetic Analysis and in Silico Antioxidant and Anti‐Inflammatory Evaluation

**DOI:** 10.1002/cbdv.71091

**Published:** 2026-03-14

**Authors:** Roukia Zatout, Ouided Benslama, Noreddine Kacem Chaouche, Wenhua Lu, Samantha C. Karunarathna, Stefania Garzoli

**Affiliations:** ^1^ Department of Microbial Biotechnology Faculty of Natural and Life Science University of Blida 1 Blida Algeria; ^2^ Laboratoire De Mycologie De Biotechnologie Et De L'activité Microbienne (LaMyBAM) Département De Biologie Appliquée Université Des Frères Mentouri Constantine Algeria; ^3^ Department of Natural and Life Sciences Faculty of Exact Sciences and Natural and Life Sciences Larbi Ben M'Hidi University Oum El Bouaghi Algeria; ^4^ Center For Yunnan Plateau Biological Resources Protection and Utilization &Ampyunnan International Joint Laboratory of Fungal Sustainable Utilization in South and Southeast Asia College of Biology and Food Engineering Qujing Normal University Qujing P.R. China; ^5^ Department of Chemistry and Technologies of Drug Sapienza University Rome Italy

**Keywords:** Antioxidant activity, Anti‐inflammatory activity, Chemical composition, Fatty acids, Molecular docking, *Pholiota gallica*, Phylogenitic tree

## Abstract

*Pholiota gallica* is a rarely documented macrofungus, and little is known about its chemical composition and biological potential. This study provides an integrated morphological, molecular, and chemical characterization of *P. gallica* collected from Constantine, Algeria, along with phylogenetic analysis and an in silico evaluation of its antioxidant and anti‐inflammatory activities. Macroscopic observations revealed caps 3–8 cm in diameter, initially viscid, with adnate to slightly emarginate gills and rust‐brown spore prints. Molecular analysis based on ITS rDNA confirmed the specimens as *P. gallica*, forming a well‐supported clade with reference strains and clearly separated from closely related species. Chemical profiling showed a predominance of esters and terpenes, with 2,2,4‐trimethylpentanediol‐1,3‐diisobutyrate (31.7%), 3‐methylapopinene (19.2%), and 2,9‐dimethyldecane (14.5%) as major components. The fatty acid profile was rich in unsaturated acids, particularly linoleic acid (44.8%) and oleic acid (33.1%), known for their antioxidant, anti‐inflammatory, and antimicrobial properties. Molecular docking studies demonstrated strong binding interactions of these compounds with XO, MPO, 5‐LOX, COX‐2, and iNOS, suggesting their contribution to the bioactivity of *P. gallica*. These findings provide a solid foundation for future experimental studies and highlight the pharmacological potential of this macrofungus.

## Graphical abstract

Integrated Morphological, Molecular, Chemical Characterization and Bioactivity of the Macrofungus *Pholiota gallica*.

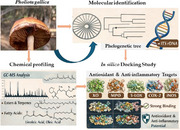

## 1 Introduction

Natural products are chemical compounds produced by living organisms and play important roles in survival, defense, and interaction with the environment [1, 2, 3]. Among these, volatile organic compounds (VOCs) and fatty acids are widely found in plants, fungi, and microorganisms. They contribute to communication, adaptation, and protection, and many of them exhibit significant antioxidant, anti‐inflammatory, and pharmacological activities, making them valuable for natural product research and therapeutic applications [4, 5, 6].

Fungi are well‐known producers of a wide range of secondary metabolites, including terpenoids, hydrocarbons, esters, alcohols, and polyunsaturated fatty acids. These compounds enhance fungal survival and ecological success and are also potential sources of bioactive molecules for pharmacological use. Compared to plants, fungal metabolites remain less explored despite their chemical diversity and biological potential. This is particularly true for macrofungi, which form visible fruiting bodies and play important ecological roles [7, 8, 9].

Macrofungi, including Basidiomycota and Ascomycota species with conspicuous sporocarps, are increasingly recognized for their nutritional, medicinal, and biotechnological value. Many species produce compounds with antioxidant, anti‐inflammatory, antimicrobial, or neuroprotective activities [10, 11]. Nevertheless, research on macrofungi is still limited worldwide, especially regarding their volatile compounds and fatty acids, which can also provide chemotaxonomic information [12].

In Africa, and especially in North African countries such as Algeria, studies on macrofungi are scarce. Most species have not been chemically characterized, and little is known about their biological properties. This is particularly true for volatile metabolites and fatty acids, which have rarely been investigated in Algerian macrofungi. Consequently, many species with ecological and potential pharmaceutical importance remain unstudied.

*Pholiota gallica* is one such species. Belonging to the family Strophariaceae, it has been poorly studied worldwide. There are no reports on its volatile compounds, fatty acid composition, or biological activities. Its molecular identity and phylogenetic placement have also not been studied in Algeria, highlighting the need for integrative studies combining morphology, molecular analysis, and chemical profiling. By contrast, other species in Strophariaceae have been more thoroughly investigated. For instance, *Stropharia rugosoannulata* has been analyzed by HS‐SPME‐GC‐MS, revealing a complex volatile profile including aldehydes, alcohols, ketones, terpenes, alkanes, and acids [13, 14]. Its fatty acid composition has also been characterized: oleic, linoleic, and eicosadienoic acids are among the most abundant fatty acids in this species [15]. Another example is *Pholiota nameko*, whose nutritional and chemical analysis showed that its lipids are rich in unsaturated fatty acids, especially linoleic acid [16]. These examples emphasize that while some Strophariaceae species are chemically profiled, *P. gallica* remains largely unexplored, making it a compelling subject for future integrative research.

The present study aims to provide the first detailed characterization of *P. gallica* from Constantine, Algeria. The study focuses on three main aspects: (i) morphological and molecular identification supported by phylogenetic analysis; (ii) profiling of volatile compounds and fatty acids; and (iii) in silico evaluation of antioxidant and anti‐inflammatory potential of the identified metabolites. This work contributes novel taxonomic, chemical, and biological insights and enriches the knowledge of North African macrofungi as potential sources of valuable natural products.

## 2 Materials and Methods

### 2.1 Collection and Morphological Identification of *P. gallica*

Fruiting bodies of *P. gallica* were collected in February 2025 by M. Bouhedja from the El Mridj Forest in the Constantine region of Algeria, where the species was found growing beneath deciduous trees. The samples were authenticated under code MC 03 based on their macroscopic features using standard taxonomic keys, and the identification was confirmed through comparison with descriptions from relevant mycological literature [17]. After collection, the specimens were washed with water and then air‐dried in the shade. The dried material was subsequently ground into a fine powder and stored for later experimental analyses [18].

### 2.2 Molecular Identification and Phylogenetic Analysis

Molecular identification was performed using a modified CTAB protocol based on Murray and Thompson [19]. A small piece of tissue (approximately 0.5 cm) was homogenized in 600 µL of 2× CTAB buffer with a micropestle and incubated at 65°C for 10 min. Next, 550 µL of chloroform was added, and the mixture was centrifuged at 13,000 rpm for 10 min. The resulting supernatant was transferred to a new tube, mixed with 450 µL of cold isopropanol, and centrifuged again for 15 min. The pellet obtained was washed with 70% cold ethanol, centrifuged for 2 min, air‐dried, and finally resuspended in 200 µL of double‐distilled water (ddH_2_O), then stored at −20°C until further analysis. PCR amplification of the internal transcribed spacer (ITS) region of ribosomal DNA was carried out using the universal primers ITS1F and ITS4 and the amplicons were sequenced using the same primers [20, 21]. The resulting sequences were deposited in GenBank [22].

The ITS sequence obtained in this study was first compared with reference sequences in GenBank using BLASTn to verify its preliminary identification [23, 24]. For the phylogenetic analysis, we compiled a dataset that included representative *Pholiota* species and related genera (*Agrocybe* and *Cyclocybe*), and we used *Cyclocybe parasitica* as the outgroup. Sequence alignment was carried out in MAFFT (version 7) with default settings [25], and regions that aligned poorly were checked and adjusted manually when needed. Phylogenetic relationships were inferred using both Maximum Likelihood (ML) and Bayesian Inference (BI). ML analysis was performed in MEGA X with the Kimura 2‐parameter model and 1,000 bootstrap replicates [26]. BI analysis was conducted in MrBayes v.3.2.7a under the GTR+G model, running four Markov chains for 2 million generations and sampling every 100 generations; the first 25% of samples were discarded as burn‐in. Bootstrap values of 70% or higher and posterior probabilities of at least 0.90 were considered strong support. The final tree was visualized and edited in FigTree v1.4.4 [27].

### 2.3 HS‐SPME‐GC‐MS Analysis of *P. gallica*

The volatile chemical composition of *P. gallica* dry powder was performed by HS‐SPME sampling technique followed by GC‐MS analysis. About 0.5 g of the powder was placed inside a 4 mL glass vial with PTFE‐coated silicone septum [28]. The extraction process of volatiles was carried out on a SPME device from Supelco (Bellefonte, PA) with 1 cm fiber coated with 50/30 µm DVB/CAR/PDMS (divinylbenzene/carboxen/polydimethylsiloxane). Before use, the fiber was conditioned at 270°C for 30 min. The equilibration time was obtained heating to 60°C for 15 min. After this time, the fiber was exposed to the headspace of the samples for 20 min at 60°C to capture and concentrate the volatiles. Lastly, the analytes were desorbed thermally in the GC injector maintained at 250°C for 2 min in splitless mode. The analysis was carried out on Clarus 500 model Perkin Elmer (Waltham, MA, USA) gas chromatograph coupled with a mass spectrometer equipped. The capillary column was a Varian Factor Four VF‐5. To characterize the volatile composition of the sample, the operative conditions were set as follows: from 45°C to 220°C at 6°/min and finally held for 15 min. Helium was used as carrier gas at a constant rate of 1 mL/min. MS scans were recorded within the range 35–450m/z using EI ionization (energy 70 eV). Identification of compounds was based on the comparison of the mass spectra of pure components stored in the Wiley 2.2 and Nist 11 libraries database and on the comparison of the Linear Retention Indices (LRIs) calculated using a series of alkane standards (C_8_–C_25_ *n*‐alkanes) with that available retention data reported in the literature. The relative amounts of the constituents were expressed as percentages (mean of three replicates) without the use of an internal standard and any factor correction.

### 2.4 Fatty Acids Analysis

Fatty acid content was determined by GC/MS technique after lipid extraction process and synthesis of FAs methyl esters. In detail, the samples (0.5 g) were dissolved with 10 mL of chloroform/methanol (2:1 v/v). Then, 1 mL of each extract was dried with nitrogen and transmethylated with BF3‐CH3OH at 70°C for 30 min. The extraction of the obtained fatty acid methyl esters (FAMEs) was carried out with n‐hexane. 2 µL of the extract was injected into the column in splitless mode. The gas chromatographic conditions were as follows: the injector was set at 280°C and the oven temperature program started from 170°C and increased up to 260°C with a rate of 3°C/min and held constant for 15 min. Component identification and quantification were performed as above described. The analyses were performed in triplicate.

### 2.5 Molecular Docking

#### 2.5.1 Ligand Preparation

The chemical structures of the bioactive compounds identified from *P. gallica* were retrieved from PubChem (https://pubchem.ncbi.nlm.nih.gov/). The structures were downloaded in SDF format and converted to the appropriate docking format using OpenBabel (version 3.1.1). Protonation states were assigned at pH 7.4, and all rotatable bonds were allowed to rotate during the docking simulations to account for ligand flexibility.

#### 2.5.2 Protein Target Preparation

The crystal structures of the selected antioxidant and anti‐inflammatory targets were obtained from the Protein Data Bank (PDB): xanthine oxidase (XO, PDB ID: 3NVY), myeloperoxidase (MPO, PDB ID: 6WY7), 5‐lipoxygenase (5‐LOX, PDB ID: 3V99), cyclooxygenase‐2 (COX‐2, PDB ID: 6COX), and inducible nitric oxide synthase (iNOS, PDB ID: 4NOS). Water molecules and co‐crystallized ions were removed. Hydrogen atoms were added, and protonation states of ionizable residues were adjusted to pH 7.4 [29].

#### 2.5.3 Molecular Docking Protocol

Molecular docking simulations were performed using MOE (Molecular Operating Environment) 2023.08 (Chemical Computing Group, Montreal, Canada). For each protein target, the active site was defined based on the location of the co‐crystallized ligand, and a site finder tool was used to confirm key residues involved in binding. Prior to docking, the co‐crystallized ligands were re‐docked to validate the docking protocol, and the resulting poses were compared to the experimental conformations, ensuring a root‐mean‐square deviation (RMSD) below 1.8 Å, which confirmed the reliability of the method [30].

All identified mushroom‐derived ligands were docked flexibly, allowing all rotatable bonds to adjust during the simulation [31]. Docking calculations were performed using the Triangle Matcher placement method, followed by London dG scoring for pose evaluation, and the top‐ranking poses were refined using the Force Field refinement protocol [32]. Post‐docking analyses were performed to evaluate hydrogen bonds (distances < 3.0 Å), hydrophobic interactions, and other key contacts within the binding site using Discovery Studio [33]. The three top‐scoring compounds for each target, based on binding energies and interaction profiles, were selected for detailed discussion.

## 3 Results and Discussion

### 3.1 Morphological Description of Macrofungus

The collected specimens were identified as *P. gallica* a species belonging to the genus *Pholiota*, within the family Strophariaceae, order Agaricales, class Agaricomycetes, and division Basidiomycota. This species has been reported under several synonyms, including *Pholiota lubrica* var. *obscura* and *Pholiota highlandensis* var. *citrinosquamulosa*, and is commonly referred to as “Pholiote de Gaule” [34, 35].

**FIGURE 1:**
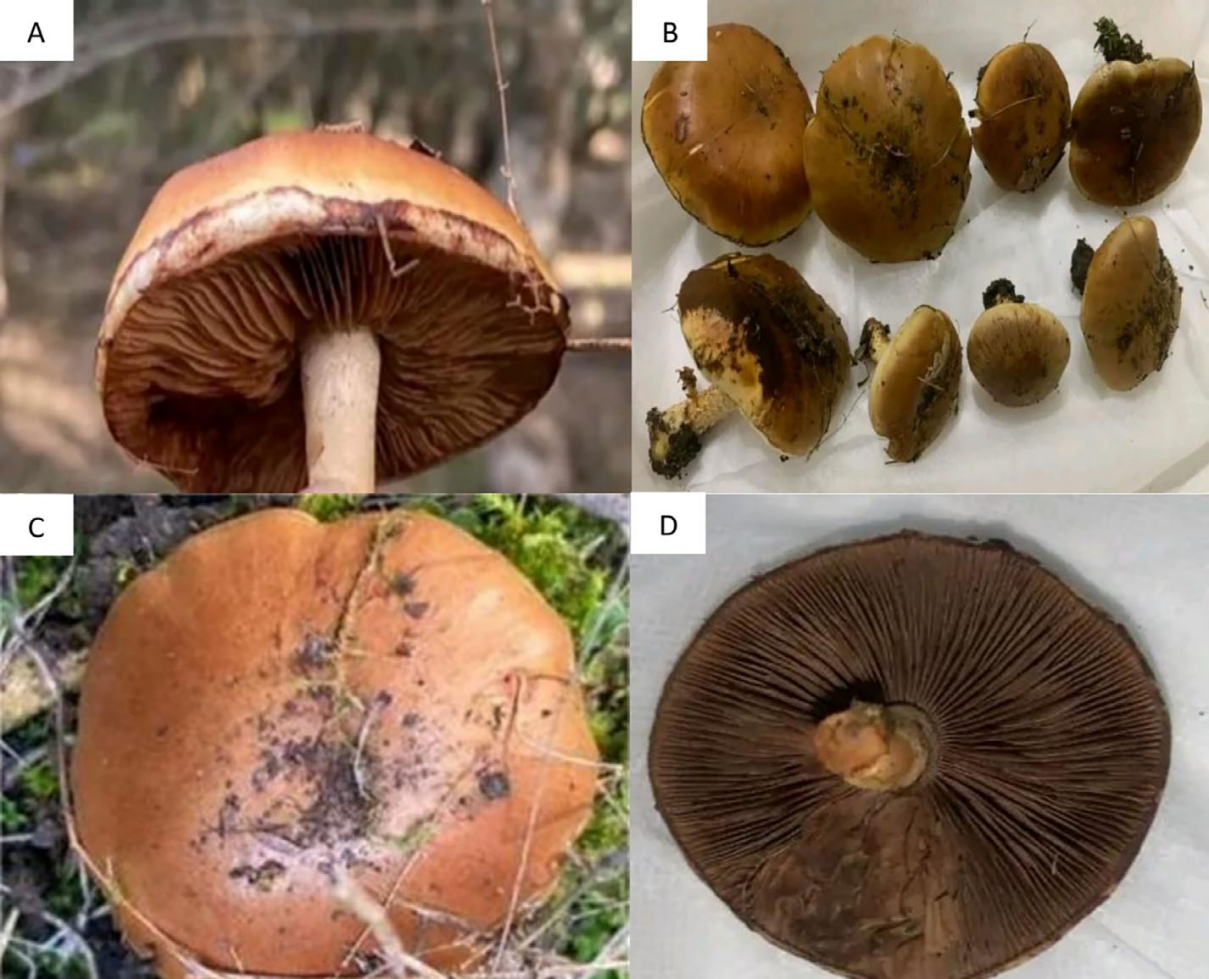
Morphological characteristics of *P. gallica* collected from Constantine, Algeria. A. Gill structure (lamellae) displaying the characteristic brown coloration. B. A collection of *P. gallica* specimens. C. Dorsal view showing the smooth brown cap surface. D. Inferior view of the fruiting body highlighting the dense lamellae and central stipe insertion.

Macroscopic examination revealed that the cap measured 3–8 cm in diameter, initially convex and ranging in color from reddish‐brown to orange‐brown, gradually becoming ochre toward the margin. Its surface was viscid when young but dried with age, while the margin of immature caps retained remnants of a white veil. The gills were adnate to slightly emarginate, moderately crowded, and changed from white to beige‐gray with a rusty tint as the spores matured. The stipe measured 4–10 cm in length and 0.5–1.5 cm in diameter, initially whitish but turning brownish at the base with age, and displayed a fibrillose to scaly surface beneath the cortina; when present, the veil was white. The flesh was pale yellow to light brown, with a mild taste and a faintly fungal or earthy odor. The spore print was rust‐brown, and the species is considered nonedible but nontoxic (Figure 1). These macroscopic characteristics were consistent with descriptions in classical mycological literature and served to confirm the identification of the specimens as *P. gallica* [34, 36].

### 3.2 Phylogenetic Analysis

**FIGURE 2:**
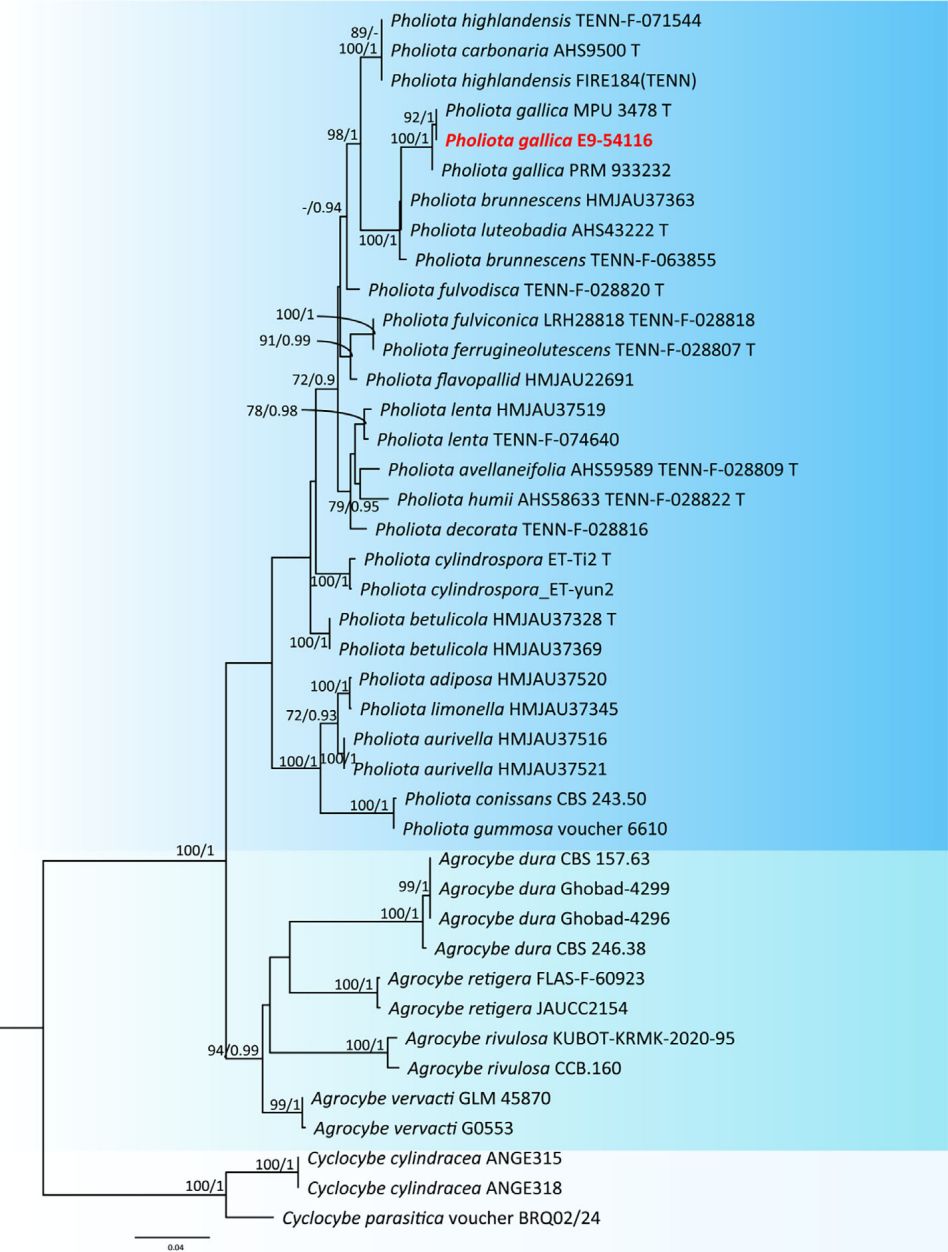
Maximum Likelihood phylogenetic tree constructed from ITS rDNA sequences illustrating the clustering of the Algerian *P. gallica* strain (EG‐54115) with reference strains and its separation from closely related species.

The ITS sequence obtained from the Algerian specimen clustered clearly within the *P. gallica* clade. In the resulting phylogenetic tree, the sequence EG‐54115 grouped with the reference strains MPU 3478T and PRM 933322, forming a well‐supported monophyletic lineage (Figure 2). High bootstrap values and posterior probabilities confirmed the stability of this clade and supported the identification of the Algerian material as *P. gallica* [37]. The *P. gallica* group appeared closely related to species of the *Pholiota highlandensis*–*P. carbonaria* complex, although the boundaries between these lineages remained well defined. In contrast, all *Agrocybe* and *Cyclocybe* sequences formed distinct clusters, clearly separated from *Pholiota*. Their placement as outgroups provided additional confidence in the overall structure of the tree. The phylogenetic results offer strong evidence that the Algerian specimen corresponds to *P. gallica*. Its close association with the reference strains MPU 3478T and PRM 933322 highlights the reliability of the molecular identification and confirms the presence of this species in the region. Although *P. gallica* shares some morphological traits with species such as *P. highlandensis* and *P. carbonaria*, the ITS‐based phylogeny shows a clear and well‐supported separation. This underlines the value of molecular tools for resolving species limits in *Pholiota*, a genus where macroscopic features often overlap.

### 3.3 Volatile Chemical Composition of *P. gallica*

The analysis of volatile compounds in *P. gallica* revealed a complex mixture of hydrocarbons, terpenes, and esters (Table 1). The major components included 2,2,4‐trimethylpentanediol‐1,3‐diisobutyrate (31.7%), 3‐methylapopinene (19.2%), 2,9‐dimethyldecane (14.5%), and 2,6,10,15‐tetramethylheptadecane (12.3%). Among these, terpenes such as 3‐methylapopinene and limonene (6.6%) were prominent and are well‐known for their antimicrobial and antifungal properties, consistent with previous studies reporting the inhibitory effects of *P. gallica* extracts against phytopathogenic fungi [38, 39]. The hydrocarbon fraction, including 2,9‐dimethyldecane and 2,6,10,15‐tetramethylheptadecane, also contributed substantially to the volatile profile. Although hydrocarbons are generally considered less bioactive individually, previous studies have suggested that complex mixtures of volatile compounds may influence biological responses [40, 41].

**TABLE 1:** Chemical volatile composition of *P. gallica* (percentage mean values±standard deviation).The components are reported according to their elution order on apolar column; Linear Retention Indices measured on apolar column; Linear Retention indices from literature.

N.	Component	LRI^calc^	LRI^lit^	%
1	3‐methylapopinene	1005	—	19.2 ± 2.15
2	limonene	1019	1023	6.6 ± 0.35
3	undecane	1120	1115	4.7 ± 0.27
4	2,9‐dimethyldecane	1128	1130	14.5 ± 1.11
5	3‐hydroxybutanal	1135	1133	1.7 ± 0.07
6	tridecane	1296	1291	2.2 ± 0.07
7	hexadecane	1615	1612	5.2 ± 0.12
8	2,6,10,15‐tetramethylheptadecane	1917	1914	12.3 ± 1.09
9	2,2,4‐trimethylpentanediol‐1,3‐diisobutyrate	1595	1591	31.7 ± 4.12
	**TOTAL**			98.1

### 3.4 Fatty Acids of *P. gallica*

The analysis of the fatty acid composition of *P. gallica* revealed a predominance of unsaturated fatty acids, with linoleic acid (44.8%) and oleic acid (33.1%) being the most abundant. Saturated fatty acids, including palmitic (19.7%) and stearic acids (2.4%), were present in smaller proportions (Table 2).

**TABLE 2:** Fatty acids content of *P. gallica* (percentage mean values ± standard deviation).The components are reported according to their elution order on apolar column (VF‐5 ms); Linear Retention Indices measured on apolar column; Linear Retention indices from literature.

N.	Fatty acids	LRI^calc^	LRI^lit^	%
1	palmitic acid, C16:0		1973	19.7 ± 1.85
2	linoleic acid, C18:2*n*6		2152	44.8 ± 5.70
3	oleic acid, C18:1*n*9		2171	33.1 ± 2.90
4	staeric acid, C18:0		2178	2.4 ± 0.12
	**SUM**			100.0
	Saturated FAs			22.1
	Unsaturated FAs			77.9

The high proportion of unsaturated fatty acids is noteworthy, given their well‐documented biological activities. Unsaturated fatty acids, such as linoleic and oleic acids, are known to possess antifungal, antimicrobial, antioxidant, and anti‐inflammatory properties [42]. Their mechanisms of action often involve disruption of microbial cell membranes, leading to increased permeability and eventual cell death [43, 44, 45].

These findings are consistent with previous reports highlighting the significance of unsaturated fatty acids in fungi as contributors to both nutritional and therapeutic properties. The relatively lower content of saturated fatty acids, such as palmitic and stearic acids, may suggest a reduced risk of pro‐inflammatory effects, further supporting the potential health benefits of this species.

### 3.5 Molecular Docking

Molecular docking was performed to explore potential interactions between the compounds identified in *P. gallica* and selected targets involved in oxidative stress and inflammation. These activities were chosen based on previous reports describing protective effects of macrofungi against oxidative damage and inflammatory processes [46, 47, 48].

For antioxidant‐related pathways, xanthine oxidase (XO, PDB ID: 3NVY) and myeloperoxidase (MPO, PDB ID: 6WY7) were selected due to their key role in reactive oxygen species production [49, 50]. For anti‐inflammatory mechanisms, 5‐lipoxygenase (5‐LOX, PDB ID: 3V99), cyclooxygenase‐2 (COX‐2, PDB ID: 6COX), and inducible nitric oxide synthase (iNOS, PDB ID: 4NOS) were chosen because of their involvement in pro‐inflammatory mediator synthesis [51, 52].

All identified compounds were docked against these targets to evaluate predicted binding affinities and interaction profiles. For clarity, only the three best‐scoring compounds for each enzyme were selected for detailed discussion.

It should be noted that docking results provide computational predictions and do not replace experimental validation. Therefore, the findings should be interpreted cautiously.

### 3.6 Antioxidant Activity

**FIGURE 3:**
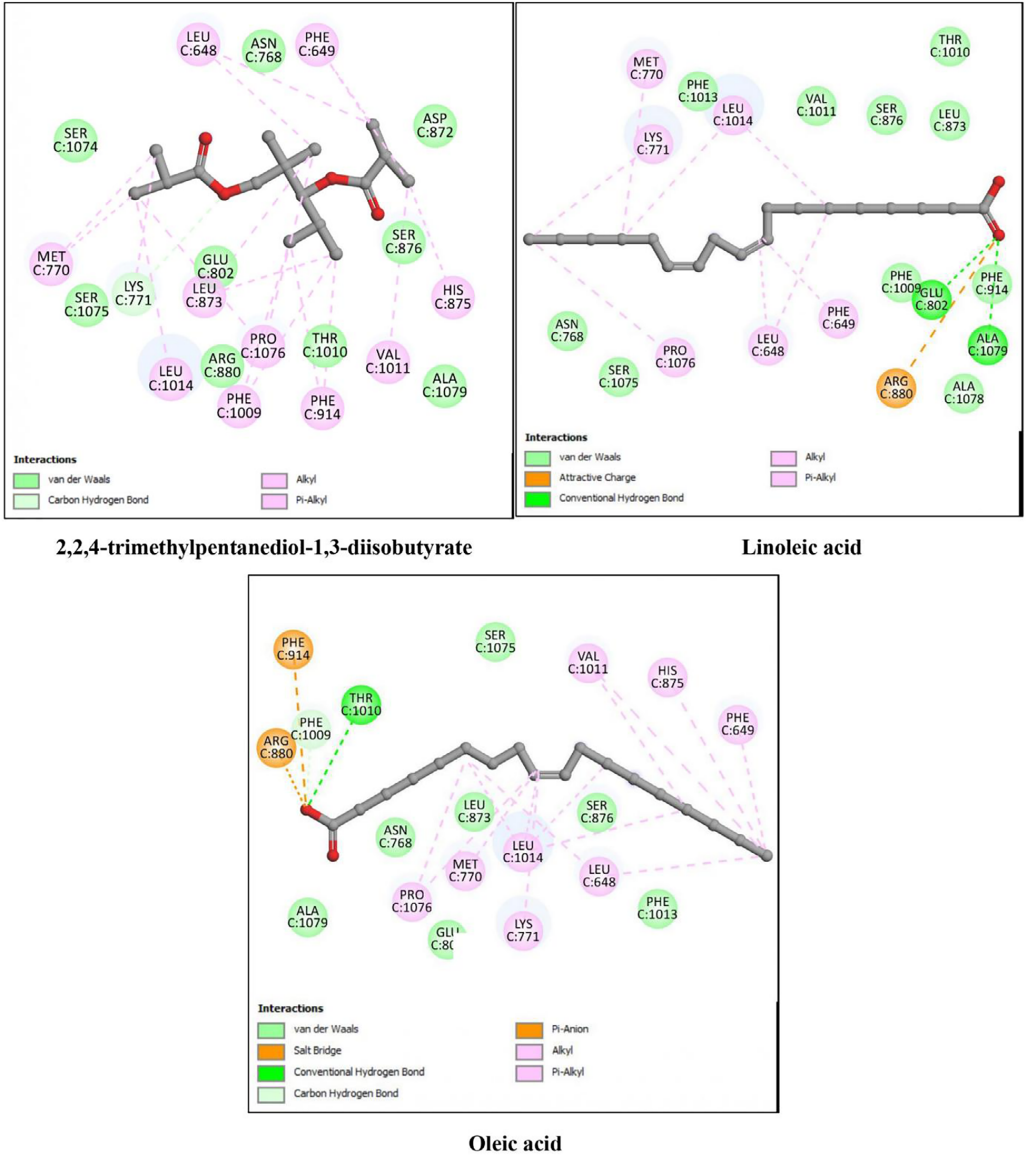
2D interaction diagrams of the top three docked ligands with xanthine oxidase (XO, PDB ID: 3NVY). The figure illustrates hydrogen bonds, hydrophobic contacts, and key active‐site residues involved in ligand stabilization.

**FIGURE 4:**
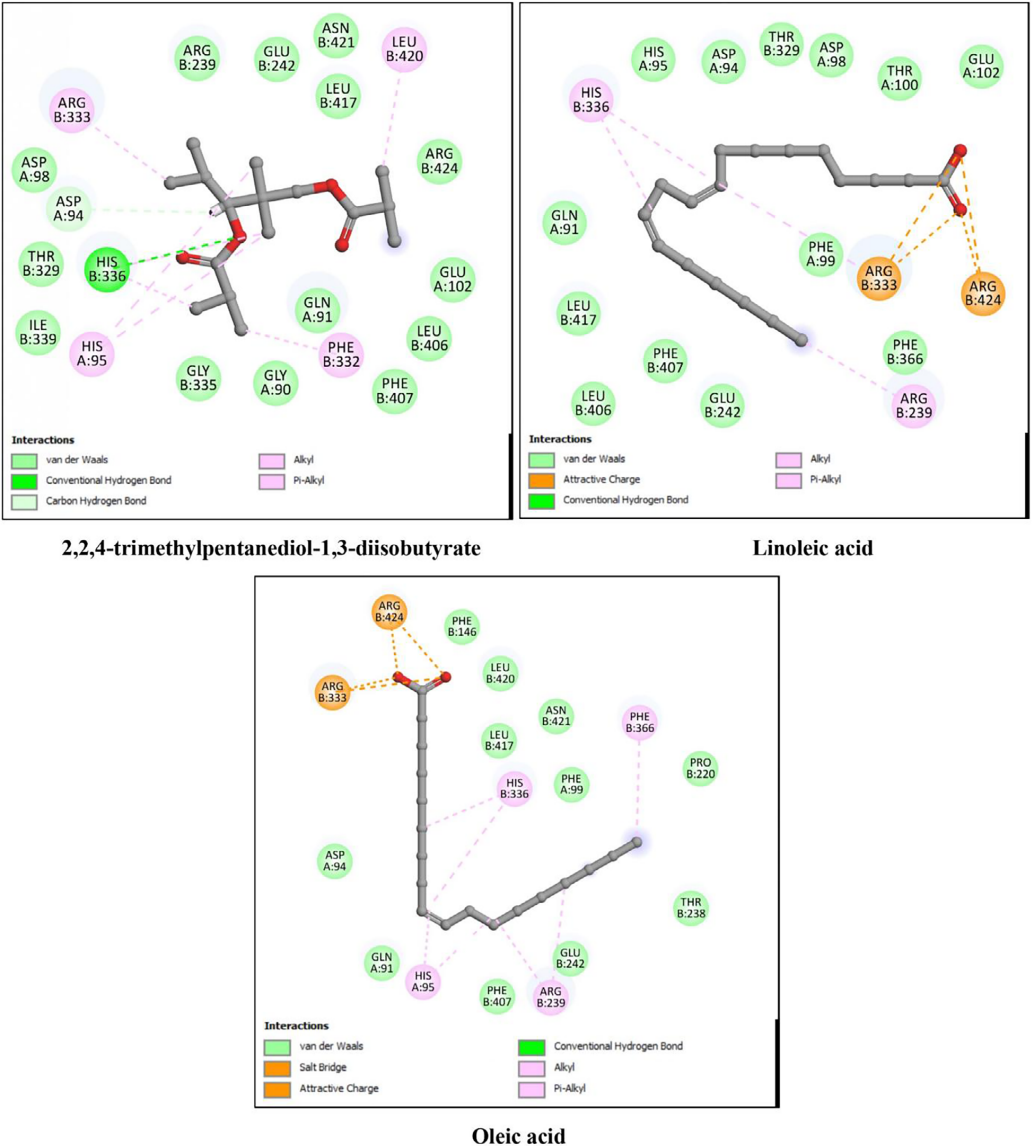
2D interaction diagrams of the top three docked ligands with myeloperoxidase (MPO, PDB ID: 6WY7). Hydrogen bonding patterns and major hydrophobic interactions with catalytic residues are highlighted.

The antioxidant activity of *P. gallica* compounds was investigated using xanthine oxidase (XO, PDB ID: 3NVY) and myeloperoxidase (MPO, PDB ID: 6WY7) as representative targets. The docking results are summarized in Table 3 and illustrated in Figures 3 and 4.

**TABLE 3:** Molecular docking results of the top three bioactive compounds from *P. gallica* against antioxidant targets xanthine oxidase (XO) and myeloperoxidase (MPO).

			Binding energy (Kcal/mol)	Hydrogen interactions (Distance Å)	Hydrophobic interactions
XO (3NVY)	Co‐crystallized ligand	Quercetin	−7.1	Asn768 (268), Glu802 (2.81), Glu802 (2.75), Arg880 (2.63), Phe1009 (3.17), Thr1010 (2.68), Ser876 (3.06)	Leu1014, Leu648
	Best docked compounds	Oleic acid	−7.0	Phe1009 (1.95), Thr1010 (2.45)	Phe649, His875, Val1011, Leu648, Lys771, Leu1014, Met770, Pro1076, Phe914, Arg880
		Linoleic acid	−6.9	Glu802 (2.62), Ala1079 (2.41)	Met770, Lys771, Leu1014, Pro1076, Leu648, Phe649, Arg880
		2,2,4‐trimethylpentanediol‐1,3‐diisobutyrate	−6.8	Lys771 (2.78)	Phe649, Leu648, Met770, Lys771, Leu1014, Leu873, Phe1009, Pro1076, Phe914, Val1011, His875
MPO (6WY7)	Co‐crystallized ligand	UFD	−8.3	Glu242 (2.65), His336 (2.55)	Leu417, His95, Leu420, Leu415, Glu102, Arg239, Glu242
	Best docked compounds	2,2,4‐trimethylpentanediol‐1,3‐diisobutyrate	−7.1	Asp92 (2.55), His336 (2.92)	Leu420, Arg333, His95, Phe332, His336
		Oleic acid	−7.1	Arg424 (2.32)	Phe366, His336, Arg239, His95, Arg424, Arg333
		Linoleic acid	−6.8	Arg424 (2.62)	His336, Arg239, Arg333, Arg424

For xanthine oxidase (XO), the co‐crystallized ligand quercetin showed a binding energy of −7.1 kcal/mol. Among the mushroom‐derived compounds, oleic acid (−7.0 kcal/mol), linoleic acid (−6.9 kcal/mol), and 2,2,4‐trimethylpentanediol‐1,3‐diisobutyrate (−6.8 kcal/mol) exhibited comparable binding affinities, with predicted hydrogen bonding and hydrophobic interactions within the active site.

For myeloperoxidase (MPO), the reference ligand UFD displayed a binding energy of −8.3 kcal/mol. The identified compounds showed slightly lower but still favorable binding energies, particularly 2,2,4‐trimethylpentanediol‐1,3‐diisobutyrate and oleic acid (−7.1 kcal/mol), followed by linoleic acid (−6.8 kcal/mol), indicating stable predicted interactions with the enzyme pocket.

Overall, the docking results suggest that these major constituents may interact with antioxidant‐related enzymes. However, these findings are based on computational predictions and require experimental validation to confirm any potential inhibitory effects.

### 3.7 Anti‐Inflammatory Activity

**FIGURE 5:**
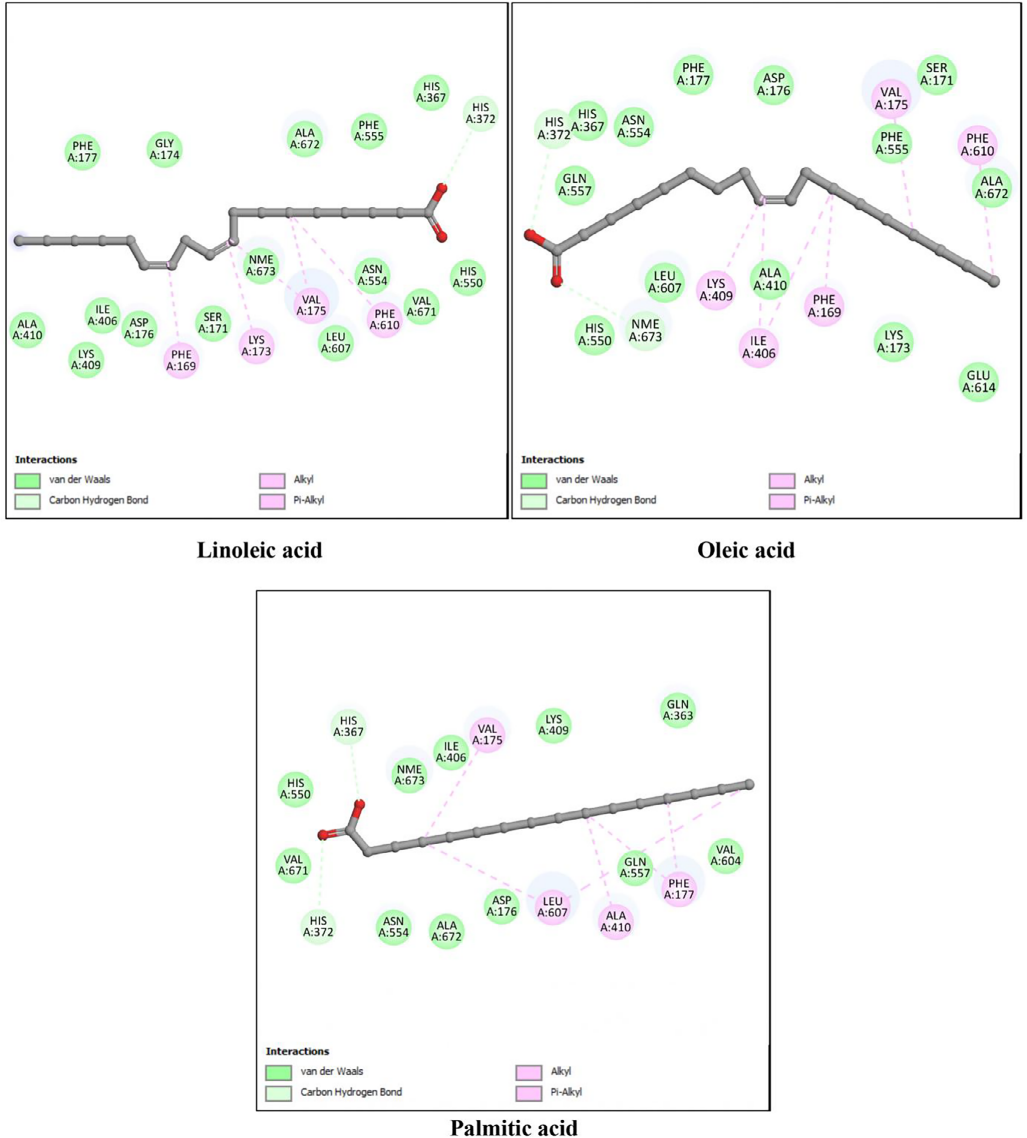
2D interaction diagrams of the top three docked ligands with 5‐lipoxygenase (5‐LOX, PDB ID: 3V99). The primary interactions within the enzyme's active site, including hydrogen bonds and hydrophobic contacts, are shown.

**FIGURE 6:**
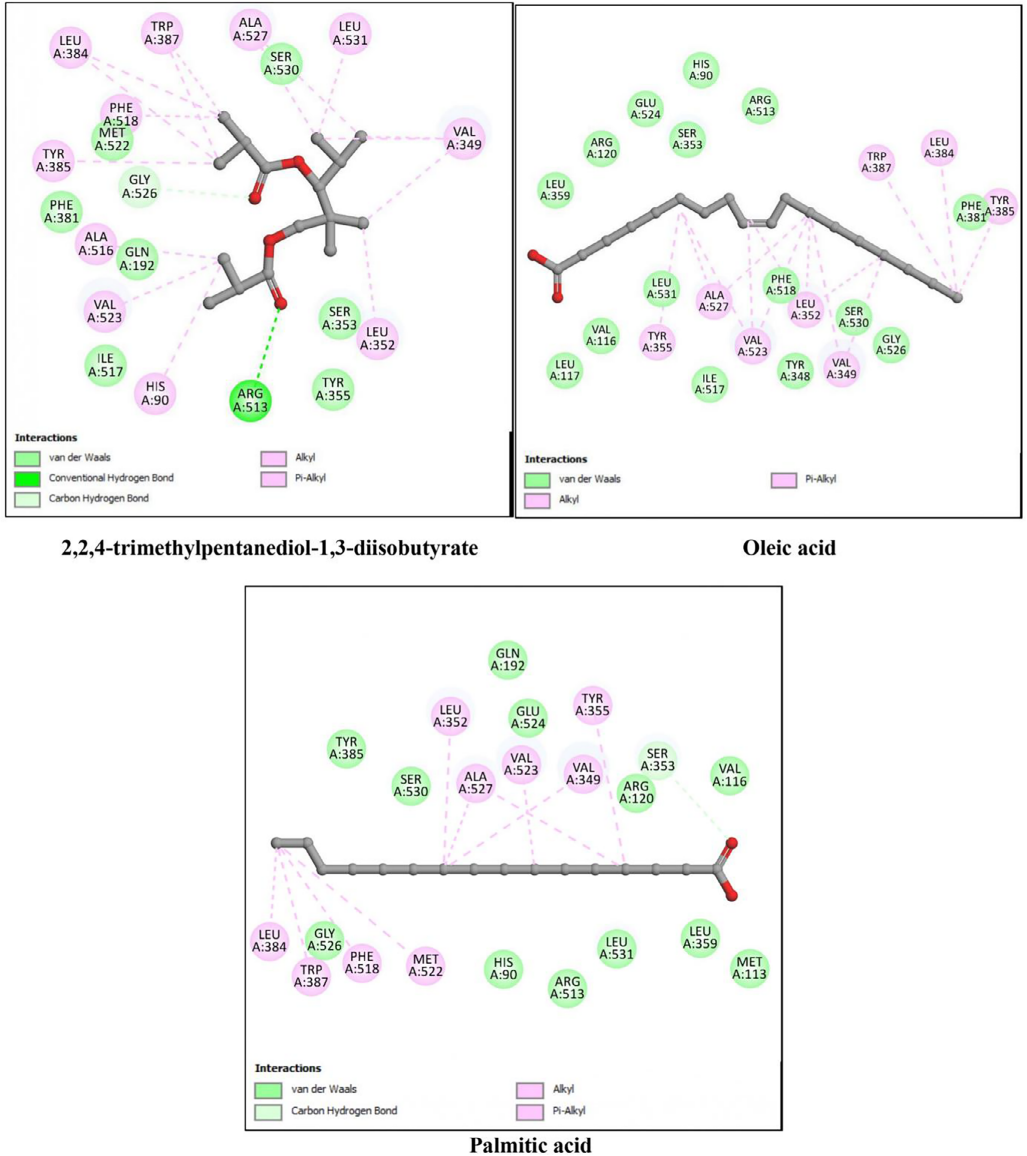
2D interaction diagrams of the top three docked ligands with cyclooxygenase‐2 (COX‐2, PDB ID: 6COX). Relevant active‐site amino acids and interaction types contributing to ligand binding are represented.

**FIGURE 7:**
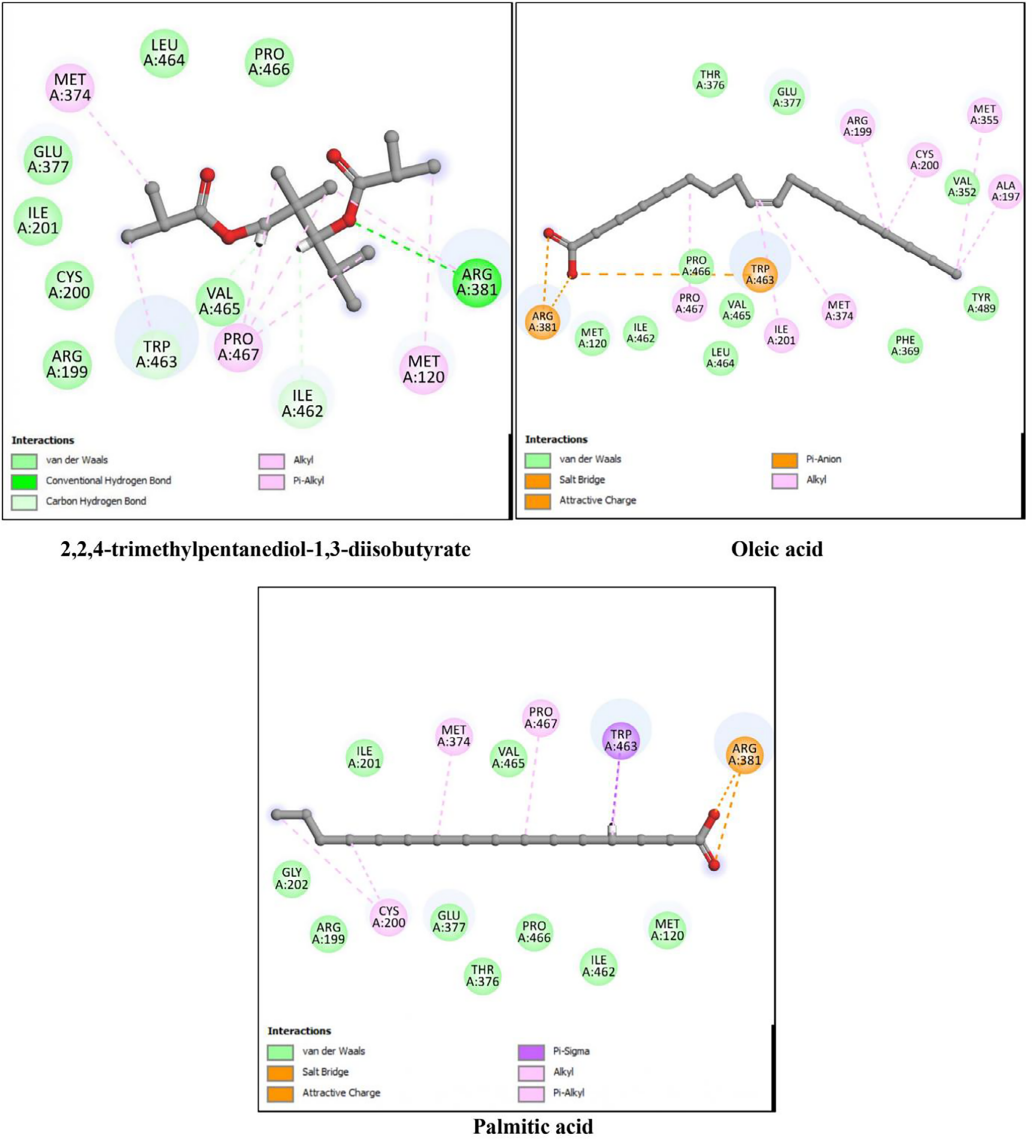
2D interaction diagrams of the top three docked ligands with inducible nitric oxide synthase (iNOS, PDB ID: 4NOS). The figure displays the hydrogen bonding network and hydrophobic interactions stabilizing ligand binding within the active pocket.

The anti‐inflammatory potential of *P. gallica* compounds was assessed using key pro‐inflammatory enzymes, namely 5‐lipoxygenase (5‐LOX, PDB ID: 3V99), cyclooxygenase‐2 (COX‐2, PDB ID: 6COX), and inducible nitric oxide synthase (iNOS, PDB ID: 4NOS). The docking results are summarized in Table 4 and illustrated in Figures 5, 6, and 7. As with the antioxidant analysis, only the three top‐scoring ligands for each target were considered for detailed discussion, although all identified compounds were initially docked.

**TABLE 4:** Molecular docking results of the top three bioactive compounds from *P. gallica* against anti‐inflammatory targets 5‐lipoxygenase (5‐LOX), cyclooxygenase‐2 (COX‐2), and inducible nitric oxide synthase (iNOS).

			Binding energy (Kcal/mol)	Hydrogen interactions (Distance Å)	Hydrophobic interactions
5‐LOX (3V99)	Co‐crystallized ligand	Arachidonic Acid	−9.1	His372 (2.52)	Phe169, Val175, Phe610, Lys173, Ile406, Lys409, Ala410
	Best docked compounds	Oleic acid	−9.2	His372 (2.66), Nme673 (2.85)	Val175, Phe610, Lys409, Ile406, Phe169
		linoleic acid	−8.9	His372 (2.46)	Phe169, Lys173, Val175, Phe610
		Palmitic acid	−7.9	His367 (2.47), His372 (2.70)	Val175, Leu607, Ala410, Phe177
COX‐2 (6COX)	Co‐crystallized ligand	S58	−9.9	Ser353 (2.33), Gln192 (2.35), Leu352 (2.41), Arg513 (3.11), Arg120 (2.52)	Leu359, Leu531, Val349, Tyr355, Val523, Phe381, Leu384, Trp387, Gly526, Tyr385, Ala527
	Best docked compounds of olive oil	2,2,4‐trimethylpentanediol‐1,3‐diisobutyrate	−8.0	Arg513 (2.85), Gly526 (2.35)	Leu352, Leu531, Ala527, Trp387, Leu384, Phe518, Trp385, Ala516, Val523, His90, Val349,
		Oleic acid	−7.8	—	Tyr385, Leu384, Trp387, Val349, Leu352, Val523, Ala527, Tyr355
		Palmitic acid	−7.8	Ser353 (2.36)	Tyr355, Val349, Val523, Ala527, Leu352, Met522, Phe518, Trp387, Leu384
iNOS (4NOS)	Co‐crystallized ligand	H2B	−6.1	Trp463 (2.35), Ile462 (2.68), Met120 (3.18), Ser118 (2.60)	Met120, Arg381
	Best docked compounds	2,2,4‐trimethylpentanediol‐1,3‐diisobutyrate	−6.1	Arg381 (3.12), Ile462 (3.16), Trp463 (2.50)	Met120, Arg381, Pro467, Trp463, Met374
		Oleic acid	−5.8	—	Ala197, Met355, Cys200, Arg199, Met374, Ile201, Trp463, Pro467, Arg381
		Palmitic acid	−5.8	—	Cys200, Met374, Pro467, Trp463, Arg381

For 5‐LOX, the reference ligand arachidonic acid showed a binding energy of −9.1 kcal/mol. Among the identified compounds, oleic acid (−9.2 kcal/mol) and linoleic acid (−8.9 kcal/mol) displayed comparable binding affinities, while palmitic acid showed slightly lower affinity (−7.9 kcal/mol). These results indicate that the major fatty acids of *P. gallica* are capable of occupying the 5‐LOX active site through predicted hydrogen bonding and hydrophobic interactions.

For COX‐2, the co‐crystallized ligand exhibited a binding energy of −9.9 kcal/mol. The mushroom‐derived compounds showed moderate binding affinities, particularly 2,2,4‐trimethylpentanediol‐1,3‐diisobutyrate (−8.0 kcal/mol), followed by oleic and palmitic acids (−7.8 kcal/mol). The interactions were mainly stabilized by hydrophobic contacts, with occasional hydrogen bonding contributions.

In the case of iNOS, the reference ligand displayed a binding energy of −6.1 kcal/mol. 2,2,4‐trimethylpentanediol‐1,3‐diisobutyrate showed comparable affinity (−6.1 kcal/mol), whereas oleic and palmitic acids exhibited slightly lower binding energies (−5.8 kcal/mol). The predicted interactions were predominantly hydrophobic.

Overall, the docking analysis suggests that oleic acid, linoleic acid, and 2,2,4‐trimethylpentanediol‐1,3‐diisobutyrate consistently showed favorable predicted affinities toward key inflammatory targets. However, these findings are based solely on computational modeling and should be interpreted cautiously pending experimental validation.

This study provides the first integrated taxonomic and chemical characterization of *P. gallica* from Algeria. While the in silico analysis offers preliminary insights into possible antioxidant and anti‐inflammatory interactions, biological activities were not experimentally validated. Future studies should include in vitro and in vivo assays to confirm the pharmacological relevance of the identified compounds.

## 4 Conclusion

*P. gallica* represents a largely unexplored macrofungus with potential biological relevance. Its chemical characterization revealed a diverse profile of volatile compounds and a predominance of unsaturated fatty acids, particularly linoleic and oleic acids, which have been reported in the literature to be associated with antioxidant and anti‐inflammatory properties. In silico docking analyses suggested that selected metabolites may interact with key enzymes involved in oxidative stress and inflammatory pathways, providing preliminary insights into possible mechanisms of action. However, these findings are based solely on computational predictions and require experimental validation. Molecular and phylogenetic analyses confirmed the identity of the species and clearly distinguished it from closely related taxa, emphasizing the importance of integrating morphological and molecular approaches in fungal taxonomy. Overall, this study provides the first integrated characterization of *P. gallica* from Algeria and establishes a scientific foundation for future investigations aimed at experimentally validating its biological activities and further exploring its chemical diversity.

## Author Contributions

**Roukia Zatout**: conceptualization, methodology, investigation, writing – original draft, supervision, review, and editing. **Ouided Benslama**: investigation, data analysis, writing – original draft. **Noreddine Kacem Chaouche**: writing – review and editing. **Wenhua Lu**: formal analysis, software. **Samantha C. Karunarathna**: formal analysis, writing – review and editing, validation. **Stefania Garzoli**: formal analysis, metodology, data curation, investigation, supervision, writing – review and editing.

## Funding

This research received no specific grant from any funding agency in the public, commercial, or not‐for‐profit sectors.

## Conflicts of Interest

The authors declare no conflict of interest.

## Data Availability

The authors have nothing to report.
